# Efforts in Organized Medicine to Eliminate Harmful Race-Based Clinical Algorithms

**DOI:** 10.1001/jamanetworkopen.2024.1121

**Published:** 2024-03-05

**Authors:** Emily C. Cleveland Manchanda, Blair Aikens, Fernando De Maio, William Jordan, Jennifer T. Brown, Karthik Sivashanker, Aletha Maybank

**Affiliations:** 1 American Medical Association, Chicago, Illinois; 2 Boston University Chobanian and Avedisian School of Medicine, Boston, Massachusetts; 3 Department of Emergency Medicine, Boston Medical Center, Boston, Massachusetts; 4 Department of Sociology, DePaul University, Chicago, Illinois

## Abstract

This survey study describes efforts to eliminate harmful race-based clinical algorithms among state or territorial medical associations and specialty societies in the US.

## Introduction

Increased attention to harmful race-based clinical algorithms—equations and decision-making tools that misuse race as a proxy for genetic or biologic ancestry^1^—has led to the reconsideration of these algorithms in many medical specialties. Most such algorithms were developed or endorsed by medical specialty societies, ensuring their widespread use.

Advocacy to eliminate the misuse of race in clinical algorithms has grown from grass-roots efforts to organized, coalition-based efforts^2^ supported by numerous medical societies. The American Medical Association (AMA) has specifically called for eliminating the misuse of race in clinical algorithms and implementing strategies to redress related harms.^3^ This call aligns with a growing movement to advance reparative approaches, which appropriately use race as a social construct, to identify and redress harms.^4^

Given the influence of medical societies in developing and legitimizing clinical algorithms, as well as anecdotal reports of societies’ efforts to eliminate harmful algorithms from use, we surveyed societies on their activities related to this and other equity issues. This article presents descriptive findings on organized medicine’s efforts to eliminate harmful race-based clinical algorithms.

## Methods

The Health Equity in Organized Medicine (HEIOM) survey (eAppendix in Supplement 1) was emailed to all Federation of Medicine organizations,^5^ collecting data from January 12 to February 28, 2023, on efforts to advance health equity. Data were collected on internal (eg, equity training for staff and leadership) and external (eg, advocacy to eliminate clinical algorithms and decision-making tools that misuse race as a proxy for genetic or biologic ancestry) actions. This survey was deemed not to be human subjects research and was therefore deemed exempt from formal review and informed consent by the institutional review board of the University of Illinois, Chicago. The study followed AAPOR reporting guidelines.

## Results

Sixty-eight organizations completed the survey, including 29 of 54 state or territorial associations and 39 of 150 specialty societies (Table). No geographic bias in responses was found; all US Census divisions were represented. Overall, 10 societies (15.6%) reported achieving advocacy objectives to eliminate algorithms and decision-making tools that incorrectly use race as a proxy for genetic or biologic ancestry; 22 (34.4%) reported working toward this (Figure). However, 12 (18.8%) had not considered taking action on this issue, and 8 (12.5%) reported it was not applicable to their organization. Similar numbers of specialty (20 [52.7%]) and territorial (9 [46.1%]) societies reported achieving or working toward elimination of these algorithms. A greater number of territorial (9 [46.1%]) than specialty (6 [15.8%]) societies were unaware of or had not considered work in this area. Successful advocacy took different forms. For example, the Medical Society of Delaware passed a resolution advocating for the elimination of race as a factor in estimated glomerular filtration rate, enabling the society to engage the statewide hospital association, local hospitals, and all labs in the state in efforts to eliminate the use of this and other harmful race-based algorithms. The American Academy of Family Physicians passed a resolution opposing the use of race as a proxy for biology or genetics in clinical evaluation and provided members with resources for opposing inappropriate use of race in medical decision-making. A full report of survey results can be found on the AMA website.^6^

**Table.** Description of the Health Equity in Organized Medicine Survey Sample

Characteristic	No. of respondents	No. of organizations in federation	Response rate, %
Total^a^	68	204	33.3
Type of organization			
Specialty society	39	150	26.0
State or territory association	29	54	53.7
Geographic distribution of state or territorial association^b^			
New England	3	6	50.0
Middle Atlantic	2	3	66.7
East North Central	4	5	80.0
West North Central	4	7	57.1
South Atlantic	5	9	55.6
East South Central	3	4	75.0
West South Central	3	4	75.0
Pacific	3	5	60.0
Mountain	2	8	25.0

^a^ Survey was also sent to 350 city and county associations; data not reported here due to low response rate for that type of organization (5.8%).

^b^ Census divisions of the United States.

**Figure.**
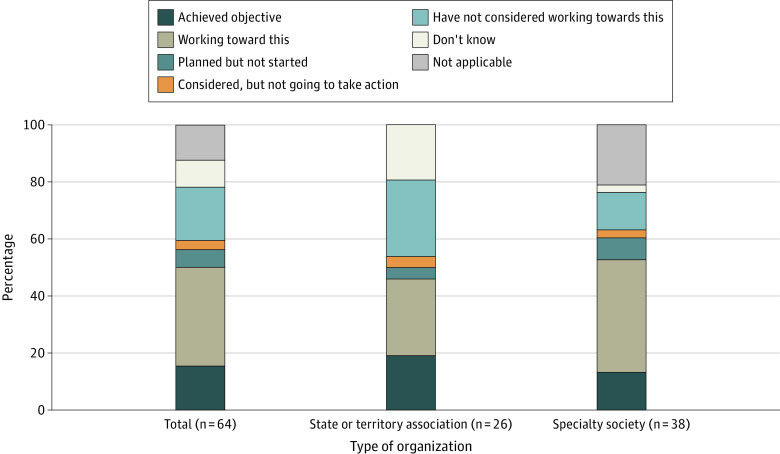
Status of State and Territorial and Specialty Medical Society Efforts to Advocate for the Elimination of Race-Based Clinical Algorithms and Decision-Making Tools That Incorrectly Use Race as a Proxy for Genetic or Biologic Ancestry Responses are missing for 4 of the total 64 organizations that did not complete this particular survey question.

## Discussion

This study serves as a baseline for accountability in organized medicine. We found notable effort to eliminate the use of harmful race-based clinical algorithms, with 50% of respondents working on this issue. Limitations include a low response rate, particularly from specialty societies, which we anticipate will improve in future years. Next steps include measuring the effectiveness of these efforts; the 2024 HEIOM survey will collect data on progress. As part of broader efforts to transform health care through coordinated and collective action, the AMA and Council of Medical Specialty Societies are convening professional societies around foundational actions to advance equity, including the elimination of harmful race-based clinical algorithms and other organizational-level contributors to inequitable access to health services.

## Notes

Supplement 1. **eAppendix.** 2023 Health Equity in Organized Medicine: Annual Association Survey

Supplement 2.
Data Sharing Statement
