# Supplementary material for: Efforts in Organized Medicine to Eliminate Harmful Race-Based Clinical Algorithms
Source: JAMA Netw Open. 2024 Mar 5;7(3):e241121. doi: 10.1001/jamanetworkopen.2024.1121 (PMC10915685; doi:10.1001/jamanetworkopen.2024.1121)
Supplement: Supplement 1. — eAppendix. 2023 Health Equity in Organized Medicine: Annual Association Survey [file jamanetwopen-e241121-s001.pdf]

Supplementary Online Content

Cleveland Manchanda EC, Aikens B, DeMaio F, et al. Progress in the elimination of harmful race-based clinical algorithms. *JAMA Netw Open*. 2024;7(3):e241121. doi:10.1001/jamanetworkopen.2024.1121

eAppendix. 2023 Health Equity in Organized Medicine: Annual Association Survey

This supplementary material has been provided by the authors to give readers additional information about their work.

© 2024 Cleveland Manchanda EC et al. *JAMA Network Open.*

eAppendix. 2023 Health Equity in Organized Medicine: Annual Association Survey

Our American Medical Association (AMA) invites you to participate in our first annual Health Equity in Medicine survey. Our objective is to understand your organization's progress towards health equity and use that understanding to identify opportunities to support your efforts.

We understand that organizations are all at different stages of learning, understanding, and taking action to address inequities in health care.

The survey will take 15-20 minutes to complete. We recommend that the survey be completed by the person or team who is most familiar with efforts to advance health equity in your organization. Please complete one survey for your organization.

Your responses will be confidential. Results shared publicly will be anonymized and shared in aggregate, so that your organization cannot be identified. We will ask in the survey on an opt-in basis if we can share any specific successes with attribution publicly.

If you have questions, please contact your Federation Relations field representative.

Thank you for your participation. These data are important for our AMA's efforts towards health equity.

Sincerely,

Aletha Maybank, MD, MPH | she|her|hers
Chief Health Equity Officer, SVP
American Medical Association

[Block A: Screening]

A01 [Required] Select your organization. If not listed, select "Another Organization" and write in your organization's name.

Drop-down list of all organizations

If another organization is selected display A02

A02 What is the name of your organization?

[Open Text]

A03 [Required] What is your title within this organization?

[open text]

A04 Are you aware of our AMA's *Organizational Strategic Plan to Embed Racial Justice and Advance Health Equity*?

1 Yes [GO TO A05]

© 2024 Cleveland Manchanda EC et al. *JAMA Network Open.*

2	No [GO TO A07]
99	Prefer not to answer [GO TO A07]

A05	My organization has used or referenced our AMA's *Organizational Strategic Plan to Embed Racial Justice and Advance Health Equity.*
1	Yes [GO TO A06]
2	No [GO TO A07]
99	Prefer not to answer [GO To A07]

A06	Please provide examples of your organization using or referencing our AMA's *Organizational Strategic Plan to Embed Racial Justice and Advance Health Equity.*
[Open Text]

A07	Are you aware of our AMA and AAMC's Advancing *Health Equity: A guide to Language, Narrative and Concepts?*
1	Yes [GO TO A08]
2	No [GO TO B01]
99	Prefer not to answer [GO TO B01]

A08	My organization has used or referenced our AMA and AAMC's Advancing *Health Equity: A guide to Language, Narrative and Concepts.*
1	Yes [GO TO A08]
2	No [GO TO B01]
99	Prefer not to answer [GO TO B01]

A09	Please provide examples of your organization using or referencing our AMA and AAMC's *Advancing Health Equity: A guide to Language, Narrative and Concepts.*
[Open Text]

[Block B: Embedding Equity]

Embedding equity into organizational culture, systems, policies, and practices only happens with intentionality and purpose.

Many organizations do this work under the name of Diversity, Equity, and Inclusion (DEI). However, the scale and type of work varies considerably across organizations.

One of several emerging frameworks for embedding equity is the competency-based Baseline Organizational Assessment for Equity Infrastructure from the California Department of Public Health.

The next few questions ask about embedding equity within your organization. The subsequent section will ask about your organization's external work.

*Throughout the survey if you hover your mouse over text in **bold**, a definition or examples will pop-up.*

© 2024 Cleveland Manchanda EC et al. *JAMA Network Open.*

B01 Does your organization have a definition of health equity, if so what is it?
[Open Text]

B02 Has your organization made health equity a strategic priority?
1 Yes
2 No
99 Prefer not to answer

B03 The root causes of health inequities include but are not limited to **racism**, **sexism**, **classism**, **homophobia**, **xenophobia**, and **ableism**. This question specifically asks about racism, and harms refer to the polices and practices that intentionally or unintentionally disadvantage historically marginalized and minoritized groups. We lead with race because history and the evidence compel us to do so. Racial inequities, representing some of the largest gaps amongst populations in this country, exist and persist in every system examined across the country.

Note: If you hover your mouse over bold text and a definition or examples will pop-up.

Indicate the actions your organization has taken to address **racism** in these systems in your practices, policies, or plans. Select all that apply.

1. Identified **harms** related to the organizations polices or practices
2. Publicly acknowledge the organization's past **harms**
3. Provide equity train to staff and leadership
4. Taken action to address past **harms** caused by the organization
5. Taken action to address contemporary **harms** caused by the organization
6. Other (write in)

B04

List any examples of your organization taking action to address other forms of oppression such as sexism, classism, homophobia, xenophobia, and ableism in your practices policies, or plans.

[Open text]

B05 The following are actions your organization can take to embed equity. For each action select the option that corresponds with your organization's current state.

© 2024 Cleveland Manchanda EC et al. *JAMA Network Open.*

	Achieved Objectives	Working towards this	Planned but not started	Considered, but not going to take action	Have not considered working towards this	Don't Know
Update bylaws to include explicit language that demonstrates the organization's commitment to health equity.						
Ensure mission, vision, and goals intentionally and explicitly address health equity.						
Evaluate how programs contribute to organizational equity goals.						
Set and align performance incentives to organizational equity goals.						
Assess your organization's budget model to ensure it will advance health equity.						
Ensure senior leadership and board members reflect the diversity of the community served by your organization.						
Commit to paying all employees and contractors a living wage.						
Collaborate with staff to revise practices and policies guiding hiring, promotion, advancement, compensation, and mediation practices to achieve equitable outcomes.						
Invest in accessible and plain-language communications, language interpretation and translation services.						
Create and/or revise incentives for staff, including the board and executive leadership, to meet organization's goals for equity, including diversification of the workforce.						
Collect and stratify key quantitative data regarding organizational leadership and staff, for relevant sociodemographic factors (e.g**. REaLD and SOGI**) to identify inequities						

Display B07 if the organization selected "achieved objective" or *"working towards" for at least one*

B07 You selected that your organization *has achieved objectives or is taking action towards* the following: [INSERT SELECTED ANSWERS]

© 2024 Cleveland Manchanda EC et al. *JAMA Network Open.*

What are some successes you have experienced while achieving or working towards this action (these actions)?
[Open Text]

Display B08 if the organization selected "Planned but not started"

B08 You selected that your organization has planned but not started the following [INSERT SELECTED ANSWERS]
What barriers, if any, might prevent you from taking this action [these actions]?
[Open Text]

Display B09 and B10 if the organization selected" Considered but not going to take action" for at least

B09 You selected that your organization has ***considered but*** ***not taken action towards*** the following [Insert selected answers]

What barriers has your organization experienced on this issue (these issues)?
[Open Text]

B10 What support or resources would your organization need to take this action (these actions)?

[Open Text]

B11 Is there anything else you would like to share with the AMA about embedding equity in your organization?

[Open Text]

© 2024 Cleveland Manchanda EC et al. *JAMA Network Open.*

Section C: External Equity Work

The previous section focused on internal actions your organization is taking to embed equity. This next set of questions asks about actions your organization is taking to advance health equity externally. This involves your work with members and beyond, including collaboration with community and patient groups.

C01 For each action, select the option that corresponds with your organization's current state. If an action is not relevant for your organization, please select "Not Applicable".

	Objective Achieved	Working towards this	Planned but not started	Considered but not going to take action	Have not considered working towards this	Don't Know	Not Applicable
Invest time in understanding your community, including assets and strengths as well as challenges that community members experience							
Gather qualitative data (e.g., individual and community experiences) to understand the full scope and context of inequities in key conditions that your organization addresses							
Publicly share equity data and indicators for transparency and mutual accountability							
Collect and stratify key quantitative data regarding membership for relevant sociodemographic factors (e.g**., REaLD and SOGI**) to identify inequities							
Address root causes of health inequities by leveraging unique organizational assets and strengths to address **social and structural drivers of health**							
Advocate to eliminate race-based clinical algorithms and decision-making tools that incorrectly use race as a proxy for genetic or biologic ancestry							
Engage in collective advocacy to address root causes of health inequities							

© 2024 Cleveland Manchanda EC et al. *JAMA Network Open.*

Display C02 if the organization selected "achieved objective" or "working towards this" for at least one action

C02 You selected that your organization has *achieved objective or is taking action towards* the following: [INSERT ACTIONS SELECTED in C01] What are some of the successes you experienced doing this work?
[OPEN TEXT]

Display C03 if the organization selected "Planned but not started"

C03 You selected your organization *has planned but not started the* following [INSERT] What barriers, if any, might prevent you from taking this action [these actions]?
[OPEN TEXT]

Display C04 and C05 if the organization selected" Considered but not going to take action" for at least one action

C04 You selected that your organization has not taken action toward the following: [INSERT ACTIONS SELECTED in C01] What is the most significant reason why your organization has not taken this action (these actions)?
[OPEN TEXT]

C05 You selected that your organization *has not taken action towards* the following [INSERT ACTIONS SELECTED in C01] What support or resources would your organization need to take this action (these actions)?
[OPEN TEXT]

Section D: Additional Topics

Below are additional topics with significant health equity implications that we would like to learn more about. For each topic indicate if your organization has initiatives or actions that you would like to share.

D01 What structural barriers to diversifying the health care workforce is your organization facing, and what possible solutions are you exploring?
[Open Text]

D02 Are there notable initiatives in your organization focusing on the **structural and social drivers of health**? If so, please explain.
[Open Text]

© 2024 Cleveland Manchanda EC et al. *JAMA Network Open.*

D03 Are there notable initiatives in your organization focusing on **maternal mortality**? If so, please explain.
[Open Text]

D04 Are there notable initiatives in your organization focusing on physician's experiences of **discrimination or violence**?
[Open Text]

D05 Are there notable initiatives in your organization focusing on **climate justice?** If so, please explain.

D06 Does your organization collaborate with **historically marginalized or minoritized physician groups** or their state or local chapters?
01 YES [GO TO C08]
02 No [GO TO C09]
03 Don't Know [GO TO C09]

D07 Which historically marginalized or minoritized physician groups does your organization collaborate with?
[Open Text]

D08 Is there anything else you would like to share about your organization's equity work?
[Open Text]

D09 There will be public sharing of organization-specific attributed success narratives and aggregated, anonymized status and challenges. Any draft document including an attributed success story will be shared with the organization for editing and approval before publication.

Do we have permission to share your organization's successes publicly (e.g., AMA website or reports)?
01 YES
02 NO

Section E: Closing

Thank you for completing the Health Equity in Medicine: Annual Association Survey. These data will be used to understand successes and challenges experienced by Federation organizations in your progress towards health equity. We will use that understanding to identify opportunities for collaboration to support your efforts.

© 2024 Cleveland Manchanda EC et al. *JAMA Network Open.*

If you agreed to share your successes publicly, we will follow-up with you. After analysis is complete, your organization will receive a summary report of this survey.

If you have any questions, please contact AMA Federation Relations.

© 2024 Cleveland Manchanda EC et al. *JAMA Network Open.*
