# Supplementary material for: Efforts in Organized Medicine to Eliminate Harmful Race-Based Clinical Algorithms
Source: JAMA Netw Open. 2024 Mar 5;7(3):e241121. doi: 10.1001/jamanetworkopen.2024.1121 (PMC10915685; doi:10.1001/jamanetworkopen.2024.1121)
Supplement: Supplement 2. — Data Sharing Statement [file jamanetwopen-e241121-s002.pdf]

Data Sharing Statement

Cleveland Manchanda. Toward the Elimination of Harmful Race-Based Clinical Algorithms. *JAMA Netw Open*. Published March 05, 2024. doi:10.1001/jamanetworkopen.2024.1121

Data
Data available: No
